# Supplementary figures and images for: Biological Control of Fusarium oxysporum f. sp. cubense Tropical Race 4 Using Natively Isolated Bacillus spp. YN0904 and YN1419
Source: J Fungi (Basel). 2021 Sep 24;7(10):795. doi: 10.3390/jof7100795 (PMC8537417; doi:10.3390/jof7100795)

# YN0904

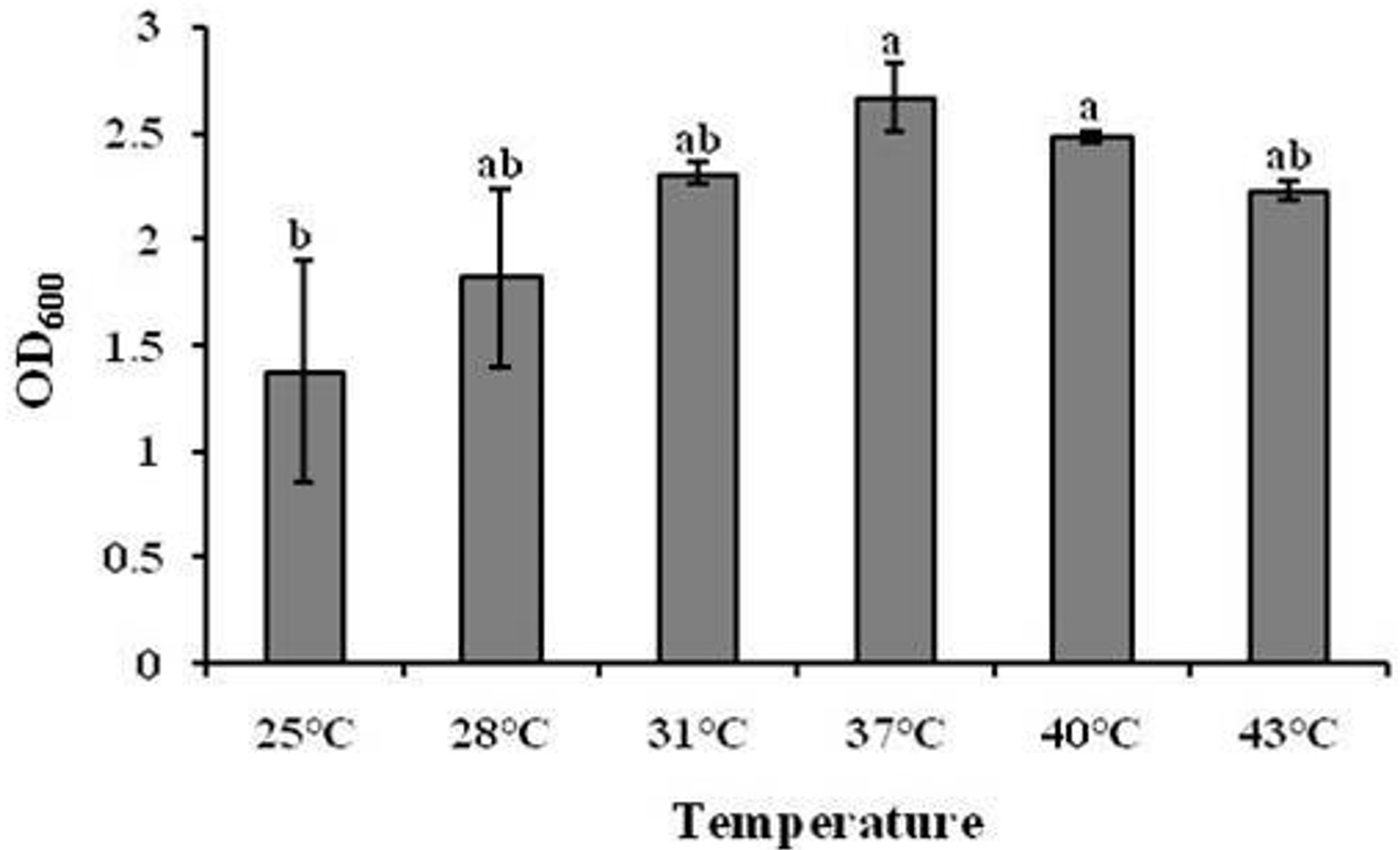

# YN1419

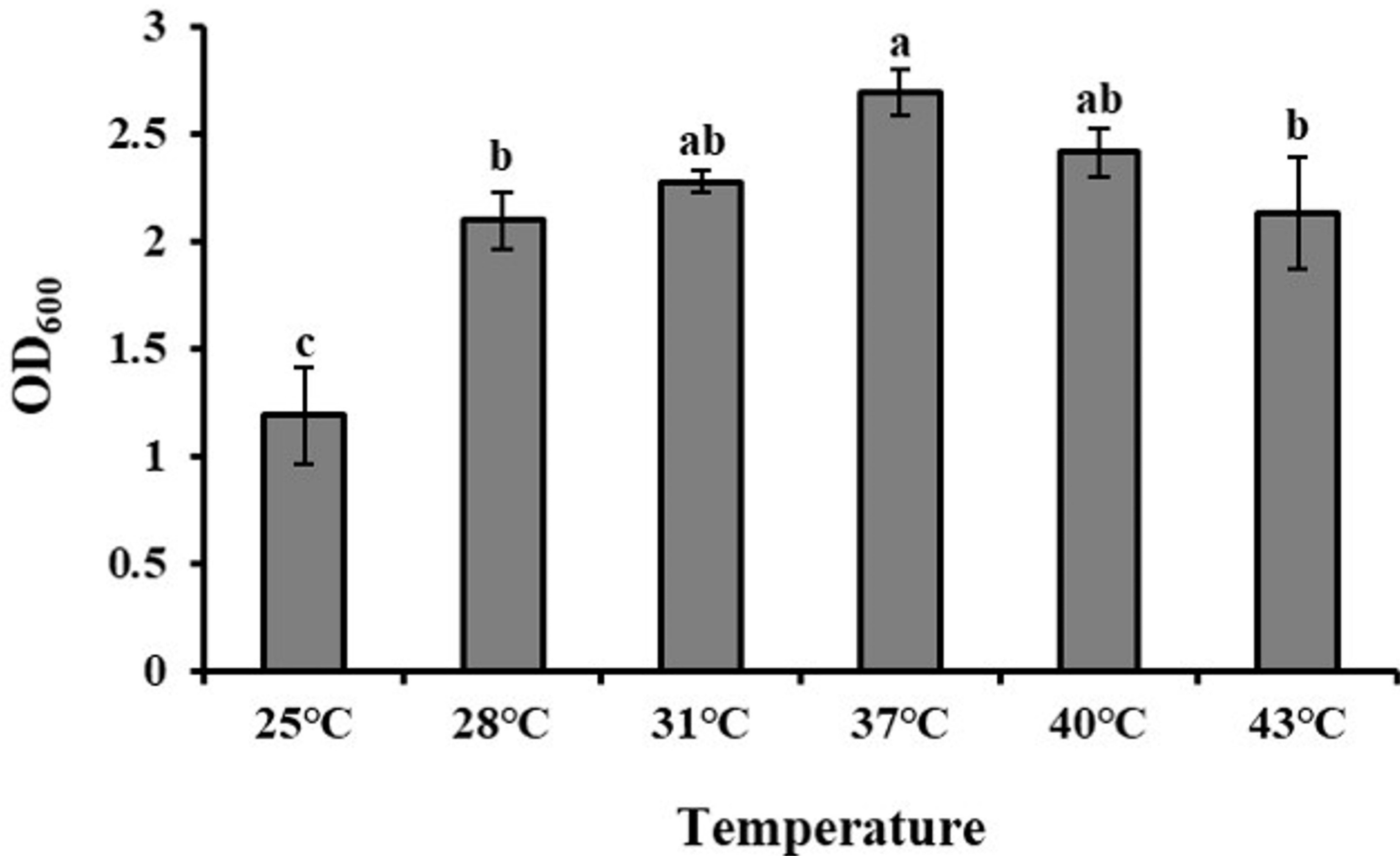

Supplement: Supplementary file 1 [file jof-07-00795-s001.zip › Figure S1. Effect of temperature on the growth rate of strains YN0904 and YN1419.pdf]

# YN0904

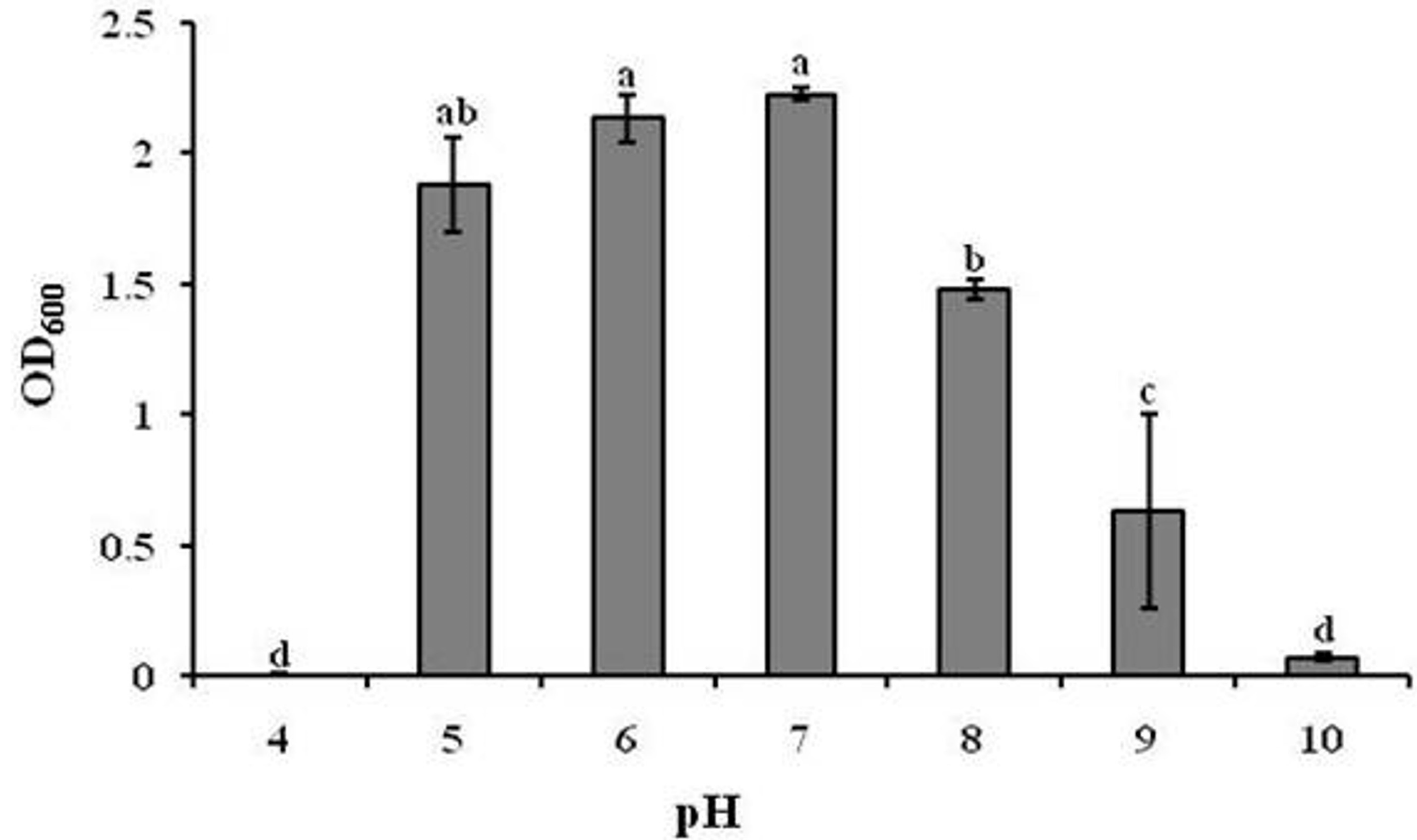

# YN1419

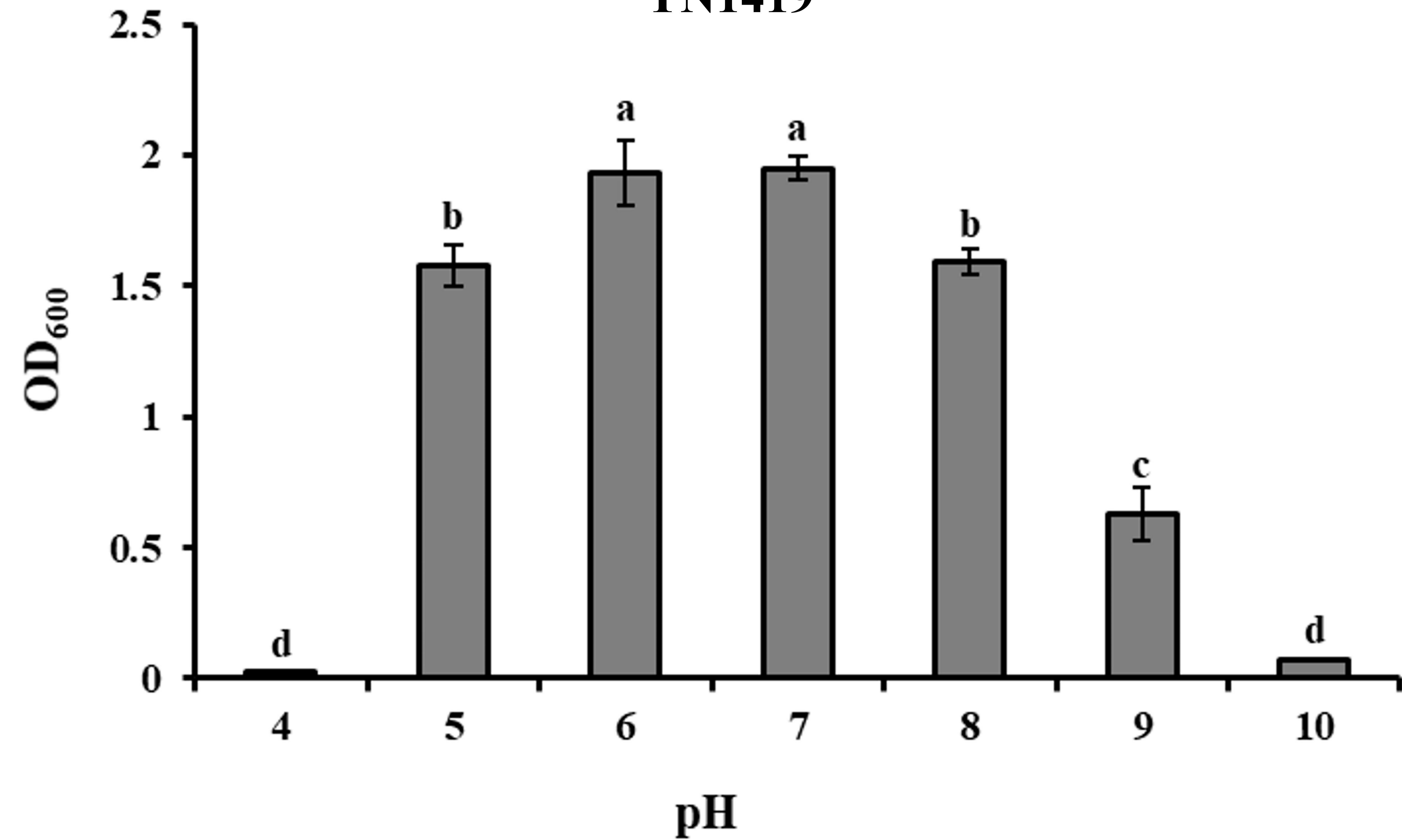

Supplement: Supplementary file 1 [file jof-07-00795-s001.zip › Figure S2. Effect of temperature on the growth rate of strain YN0904 and YN1419.pdf]

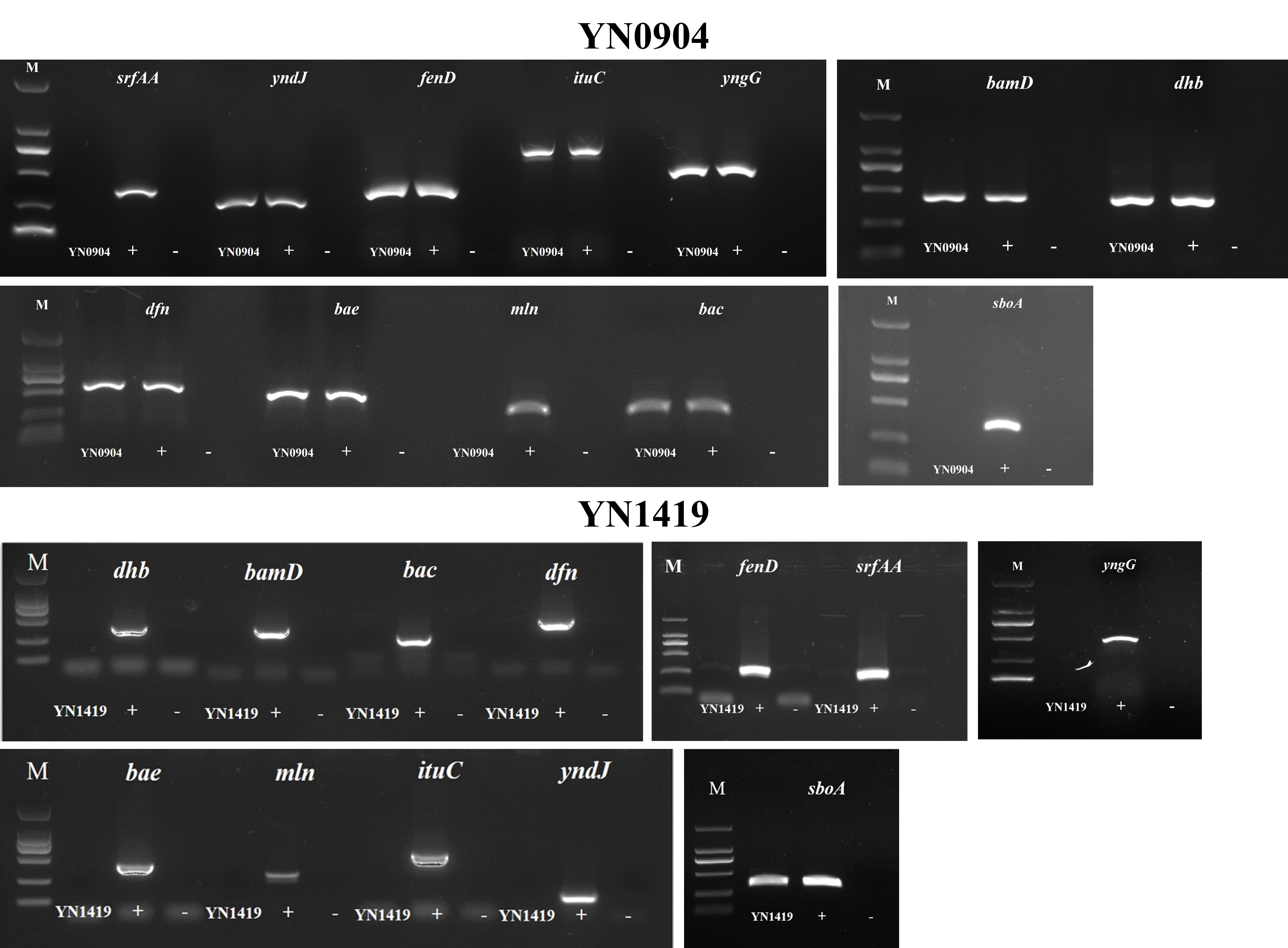

Supplement: Supplementary file 1 [file jof-07-00795-s001.zip › Figure S3.YN0904,YN1419-NRPS,PKS,RPS.jpg]

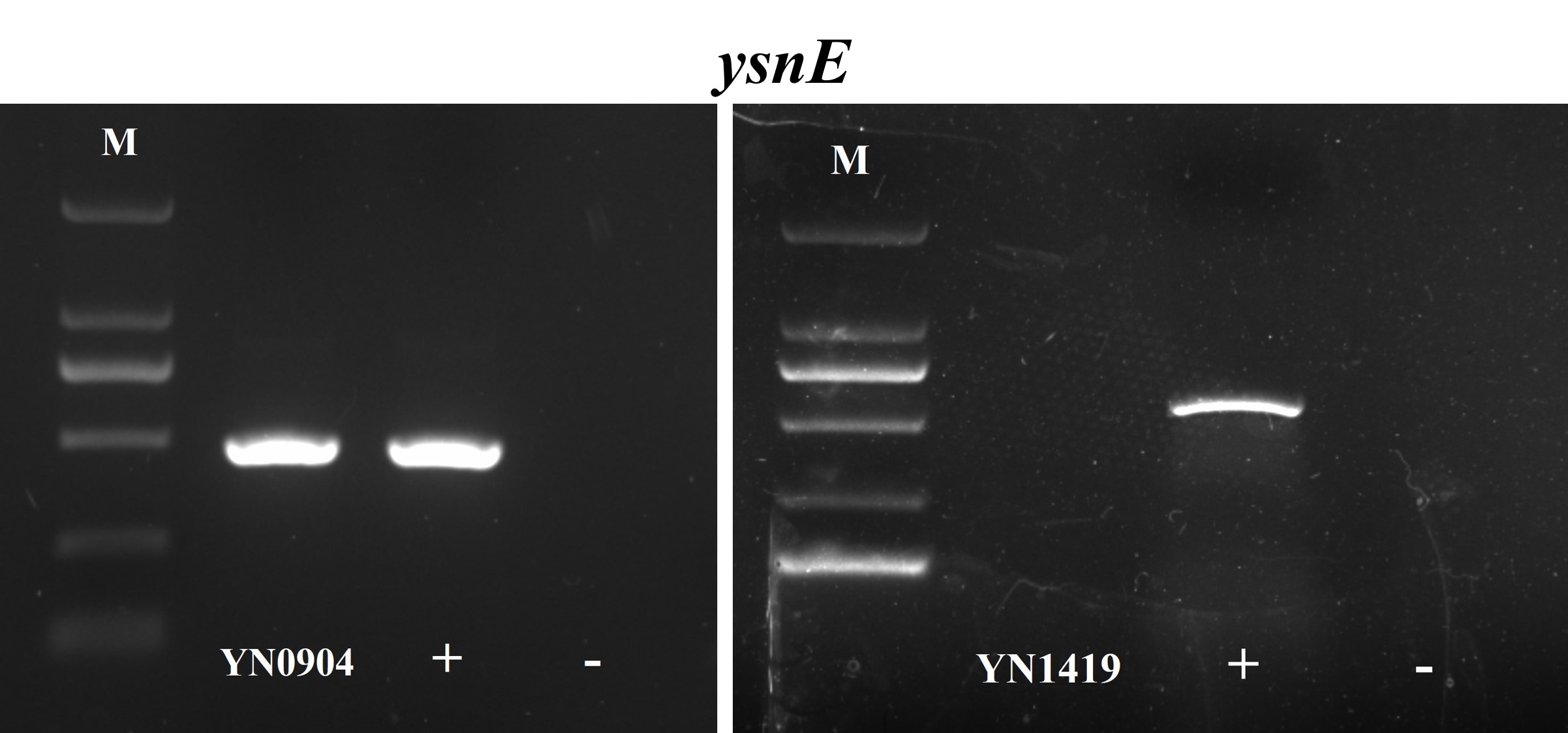

Supplement: Supplementary file 1 [file jof-07-00795-s001.zip › Figure S4.YN0904,YN1419-ysnE.jpg]
